# Oncogenic Mutations and the Tumor Microenvironment: Drivers of Non-Small Cell Lung Cancer Progression

**DOI:** 10.3390/cancers17050853

**Published:** 2025-03-01

**Authors:** Achilleas G. Mitrakas, Christos Kakouratos, Ioannis Lamprou, Erasmia Xanthopoulou, Michael I. Koukourakis

**Affiliations:** Department of Radiotherapy/Oncology, University Hospital of Alexandroupolis, Democritus University of Thrace, 68100 Alexandroupolis, Greece; ckakouratos@gmail.com (C.K.); ilamprou@med.duth.gr (I.L.); erxantho@med.duth.gr (E.X.)

**Keywords:** NSCLC, mutations, immunotherapy, stem cells, TME, TMB, lung cancer

## Abstract

Background/Objectives: Non-small cell lung cancer (NSCLC) is a major cause of cancer-related deaths globally. The study focuses on understanding the interplay between genetic mutations, cancer stem cells (CSCs), and the tumor microenvironment (TME) in driving NSCLC progression, resistance to therapies, and relapse. Methods: A systematic search was conducted in PubMed and Scopus databases to identify significant and valuable studies relevant to NSCLC, focusing on genetic mutations, CSCs, and the TME. Articles were selected based on their relevance, methodological severity, date of publication, and scientific soundness related to NSCLC biology and therapeutic strategies. This review synthesized findings from these sources to highlight key mechanisms and potential therapeutic interventions. Results: Mutations in critical genes in *KRAS*, *EGFR*, *TP53*, and other key genes interfere with stem cell regulation, promoting CSC-like behavior, resistance to therapy, and immune evasion. The tumor microenvironment (TME), including immune cells, fibroblasts, and extracellular matrix components, further supports tumor growth and reduction in treatment efficacy. Promising strategies, including CSC targeting, TME modulation, and the development of novel biomarkers, have shown potential in preclinical and clinical studies. Conclusions: The association between genetic alterations, CSCs, the TME, and other cellular pathways—including cell metabolism and immune evasion—plays a crucial role in therapy resistance, highlighting the need for comprehensive treatment strategies. The combination of genomic profiling with TME-targeting therapies could lead to personalized treatment approaches, offering hope for better clinical outcomes and reduced mortality in NSCLC patients.

## Simple Summary

Lung cancer, particularly non-small cell lung cancer (NSCLC), remains a leading cause of cancer-related deaths worldwide due to its complexity and resistance to therapy. This review explores the critical role of stem cells in lung cancer progression, focusing on how genetic mutations and the tumor microenvironment drive cancer stem cell activity, leading to therapy resistance and tumor relapse. By understanding these mechanisms, the research aims to identify potential therapeutic targets and strategies to improve treatment outcomes for NSCLC patients. This work presents the latest developments of novel personalized therapies and enhances the effectiveness of existing treatments, offering new insights and information for patients and the broader research community.

## 1. Introduction

Lung cancer is a major public health concern, as it constitutes the leading cause of cancer-related deaths in both men and women worldwide. In 2020, lung cancer was responsible for over 1.8 million deaths globally [1]. In 2023 over of 230,000 people were diagnosed with lung cancer in USA, while it is estimated that 1 in 16 men and 1 in 17 women will be diagnosed with this type of cancer in their lifetime [2]. Tobacco smoking is the main risk factor for lung cancer development. However, 20% of lung cancer deaths are not related to smoking. In addition, secondhand smoke, random gas, metals, radiation, air pollution are some of the other known risk factors, while genetic factors and family history may also play pivotal roles in cancer development [3]. Lung cancer is typically categorized in two main types, non-small cell lung cancer (NSCLC) and small cell lung cancer (SCLC). NSCLC is more common, accounting for approximately 80% of lung cancer cases, and there are three subtypes of NSCLC: adenocarcinoma, squamous cell carcinoma, and large cell carcinoma. Regarding SCLC, there are two subtypes, the small cell carcinoma and mixed small cell/large cell cancer [4].

## 2. Stem Cell Dynamics in Lung Health and Disease

In healthy lungs, stem cells or progenitor cells normally repair and regenerate the lung tissue. Alveolar type II (AT2) cells, bronchioalveolar stem cells (BASCs), and basal cells are different types of stem cells that are responsible for maintaining epithelial homeostasis. However, when these cells acquire genetic mutations, they can undergo malignant transformation, leading to uncontrolled proliferation and tumor formation. While CSCs significantly contribute to therapy resistance, tumor relapse, and self-renewal, a variety of other mechanisms, such as genetic instability, metabolic plasticity, and resistance to immune response, also play key roles. A comprehensive therapeutic approach must consider all these factors. 

### 2.1. Alveolar Type II (AT2) Cells

Alveolar type II cells are cuboidal secretory cells that serve as the defender of alveoli. These cuboidal cells are characterized by numerous surface microvilli and cytoplasmic lamellar bodies that contain a high concentration of surfactant proteins. These surfactant proteins help preserve the structure and prevent collapse of the alveoli. Pulmonary surfactant, a complex mixture of lipids and proteins, play a crucial role in maintaining the structure of the alveoli, and preventing their collapse helps prevent the alveoli from collapsing by lowering surface tension at the air–liquid interface. It includes two hydrophilic lectins known as SP-A and SP-D. AT2 cells are, also, considered progenitor cells for the alveolar epithelium, with high metabolic rates and the ability to produce a variety of components like collectins, anti-microbial, and anti-inflammatory substances [5].

These types of cells have attracted researchers’ interest for their role in tumorigenesis, particularly in the development of adenocarcinoma, one of the most common subtypes of NSCLC [6]. The reason for tumorigenesis seems to be the uncontrolled proliferation of these cells due to their malignant transformation caused by accumulation of various mutations. Adenocarcinoma is, typically, derived from cells in peripheral lung regions and is associated with mutation in the Kras gene, Egfr gene, and deletion of the tumor suppressor p53 [7,8]. It is estimated that the Kras mutations are responsible for up to 7% of all cancer mortality. KrasG12D presents oncogenic activity and is involved in AT2 transformation into growing monoclonal tumors [9]. Moreover, alterations in EGFR pathway stimulate the AT2 cells proliferation in vitro [10].

The association of pulmonary surfactant collectins with lung cancer has also attracted attention. It has been shown that patients with NSLC have a higher concentration of SP-A than patients with non-malignant conditions, while these higher levels indicate an increase in M2 suppressive macrophages and a decrease in M1 macrophages [11]. SP-D, a useful biomarker for early risk in smokers, is associated with longer progression-free survival in patients with NSCLC [12]. Both collectins can suppress the EGFR-mediated signaling [13].

Overall, the transformation of AT2 cells due to oncogenic mutations plays a central role in the development and progression of lung adenocarcinoma, making them critical for understanding lung cancer biology and developing novel targeted therapies.

### 2.2. Bronchioalveolar Stem Cells (BASCs)

Bronchioalveolar stem cells (BASCs) constitute a specific subpopulation of stem cells located at the bronchioalveolar duct junction in the lungs. They are multipotent stem cells capable of differentiating into AT2 cells and Clara cells (nonciliated secretory cells present within the bronchiolar epithelium of the lung). These cells are crucial for the normal function and regeneration of the lung’s alveolar and airway epithelium. It is supported that they play a pivotal role in repair and regeneration of lung tissue exposed to inflammation and toxic agents [14].

However, many researchers suggest their implication in lung cancer development, especially in NSCLC [15]. Similarly to AT2 cells, key mutations have been observed in significant oncogenes, such as Kras, Egfr, and TP53, that disrupt normal signaling pathways, leading to uncontrolled cells’ proliferation. Moreover, the tumor suppressor gene, pten, has been reported to be essential for normal lung morphogenesis and the prevention of cancer development [16]. Kras mutations induce the MAPK/ERK pathway driving abnormal cell division. The combination of these mutations offers high glycolytic activity and promotes cell proliferation [17]. PI3K/AKT and JAK/STAT pathways are activated by EGFR and its mutations increase BASCs’ survival and proliferation [18].

At a molecular level, several critical pathways are involved in the transformation of BASCs into cancerous cells. The Wnt/β-catenin pathway plays a significant role in stem cell renewal, and it is often overactivated in BASCs, enhancing their tumorigenic potential [19]. Additionally, the activation of Sonic Hedgehog (SHH) signaling was observed to promote the expansion of BASCs and enable them to initiate tumor growth. This pathway could provide a target for the development of prognostic markers, although further investigation is needed [20].

DNA damage and genetic mutations due to environmental factors make BASCs an important source of lung cancer stem-like cells, contributing to tumor initiation, progression, and, possibly, resistance to different therapies.

### 2.3. Basal Cells

Basal cells represent the third category of lung stem cells, which are located in the airway epithelium, particularly in the trachea and bronchi. These cells are able to regenerate the airway lining, since they serve as progenitor cells, processing the activity of self-renewal and can differentiate into other cell types in the lung, like secretory and ciliated cells that are essential for the physiological structure and function of the respiratory airways [21,22]. In a normal lung function, these types of cells seem to have a pivotal role in the epithelium repair after injury or inflammation. Their role as stem cells is essential to maintain the balance of cell populations in the airway [23].

However, basal cells may undergo genetic mutations and lose their regulatory stem cell balance, which offer unrestricted proliferation loss of differentiation into functional airway cells. TP53, Kras, Κeap1, and NOTCH1 are the main genes known to be involved in this malignant transformation and tumor development.

Specifically, mutations in TP53, a tumor suppressor gene, prevent basal cells from undergoing apoptosis or cell-cycle arrest in response to DNA damage, allowing the accumulation of further mutations that drive cancer development. It is observed that Keap1 deletion promotes tumor aggressiveness and resistance while Keap1/NRF2 could serve as novel biomarker to predict the efficacy of therapeutic strategies. Keap1 (Kelch-like ECH-associated protein 1) participates in NRF2 regulation, a transcription factor related to antioxidant response [24].

At the molecular level, similarly with the transformation of other lung stem cell pathways, EGFR, PI3K/AKT, and Wnt are commonly altered and dysregulated in basal cells, facilitating their transformation into cancerous cells. Changes in Notch signaling, another pathway essential for maintaining basal cell identity, have also been involved; aberrant Notch activation in basal cells can lead to disrupted cell differentiation and increased stem cell-like properties, further supporting NSCLC development. Together, these mutations and pathway disruptions in basal cells facilitate the progression of NSCLC and contribute to tumor heterogeneity, making these cells a potential target for therapeutic intervention [25].

## 3. Mutational Burden—Implications for Cancer Risk and Early Detection

Age and exposure to other environmental factors, for example, tobacco smoke and air pollutants, lead to an accumulation of mutations in lung stem cells. This exposure damages DNA and mutation in cancer-related genes may occur [26,27]. There are studies suggesting that a threshold of mutational burden could be identified, beyond which the possibility of malignant transformation increases. The Tumor Mutational Burden (TMB) can be assessed as the number of mutations within the DNA of tumor per megabase of genetic material. A direct association of high TMB and exposure to tobacco smoking has been observed, while there is a dose-dependent association between quantitative smoking history and TMB [28]. Moreover, high microsatellite instability (MSI-H) and mismatch repair deficiency (MMR-D) contribute to the transformation of normal cells to cancer cells leading to high TMB. MSI-H and MMR-D are associated with smoking [29].

The main query is whether the knowledge of specific mutations could contribute to improved management of NSCLC. Many studies suggest that a high mutational rate in lung stem cells can serve as an early marker for assessing cancer risk, even before tumor formation. Moreover, monitoring high-risk individuals, such as long-term smokers, can play a crucial role in developing personalized cancer prevention strategies [30]. The detection of specific mutations in key genes, combined with CSC persistence, immune suppression, and metabolic alterations, contribute to therapy resistance, and it may facilitate early intervention and aid in designing a personalized treatment method for NSCLC patients [31,32]. Addressing these interconnected factors is critical for overcoming NSCLC treatment failure

## 4. Methods for Mutational Burden Detection

Several methods are widely used to detect mutations, whether for assessing an individual’s risk of developing NSCLC or for characterizing tumors in patients. Next-Generation Sequencing (NGS) constitutes a cornerstone method for high-throughput analysis of multiple mutations simultaneously, providing a mutation profile that can be used to assess an individual’s risk and design appropriate treatment methods. Moreover, Whole-Exome Sequencing (WES), a form of NGS, focuses specifically on protein-coding regions, offering a balance between cost and mutation detection, and aiming to detect mutations in the most relevant genes found in NSCLC [33,34,35]. Additionally, immunohistochemistry (IHC) and fluorescence in situ hybridization (FISH) can also be utilized for mutation detection in NSCLC. For detecting specific, risk-related and well-characterized mutations, such as EGFR and ALK, Polymerase Chain Reaction (PCR) can be utilized; however, it falls short in providing a broad and the comprehensive genomic coverage that NGS offers [36].

Many research groups, taking into consideration the difficulty of receiving samples for the aforementioned methods, have investigated non-invasive methods to perform experimental procedure in detecting mutations. In liquid biopsies, one of the more promising methods, cell-free DNA (cfDNA) from blood samples to detect circulating tumor DNA (ctDNA) are used to monitor mutations over time, identifying genetic changes correlated to increased risk of NSCLC [37].

## 5. Stem Cells Detection in Tissues—Histochemical Insights and Prognostic Value

**Figure 1:**
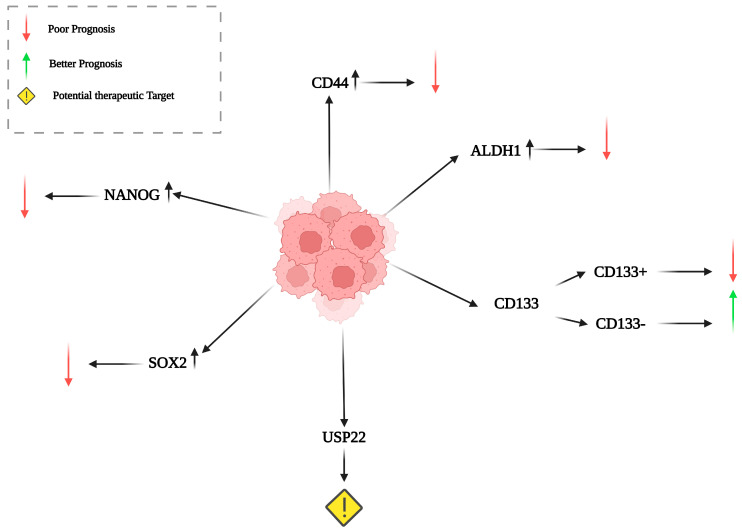
Significant immunohistochemical markers for stem cells have been identified in NSCLC. Increased levels of ALDH1A and CD44 are indicative of a more aggressive disease phenotype, associated with greater metastatic potential and poorer outcomes for NSCLC patients. CD133 is commonly recognized as a marker for cancer stem cells in lung cancer, with studies showing that individuals who are CD133-positive across various cancer types tend to have significantly worse prognoses compared to their CD133-negative counterparts. Similar findings have been reported in NSCLC patients. The presence of ALDH1A, especially in conjunction with elevated CD133 levels, correlates with a poorer prognosis. Additionally, the heightened expression of SOX2 and NANOG in NSCLC is linked to increased tumor aggressiveness, therapeutic resistance, and adverse prognostic outcomes. Recently, USP22 has emerged as a potential therapeutic target due to its oncogenic properties, with ongoing efforts to develop new inhibitors aimed at improving the effectiveness of existing treatments and enhancing patient prognoses in NSCLC (The black arrow (↑) indicates the overexpression of the specified markers).

Histochemical studies can identify specific markers to detect different types of stem cells, including cancer stem cells (CSCs) that may contribute to cancer development, progression, and metastasis (Figure 1). IHC, a traditional diagnostic and experimental method, plays a pivotal role in NSCLC management and assessment, as a proportion of NSCLC patients experience a recurrence after undergoing curative tumor resection, even those diagnosed at an early stage of the disease. Thus, characterizing the percentage of each stem cell type within NSCLC tissues can reshape therapeutic strategies. Key stem cell markers in NSCLC include (Figure 1):

### 5.1. ALDH1 (Aldehyde Dehydrogenase 1)

ALDH1 is a detoxifying enzyme that works by regulating vitamin A oxidation. Its expression levels may assist in discriminating normal cells from tumor stem cells in different types of cancer [38]. ALDH1 is involved in stem cell differentiation and self-renewal. High ALDH1 activity is associated with enhanced stem cell-like properties. ALDH1A-positive NSCLC cells present higher aggressiveness, making them a significant point for understanding tumor biology and tumor resistance to therapy. Its expression levels in combination with higher levels of CD133 are linked to poorer prognosis [39,40].

### 5.2. CD133 (Prominin-1)

CD133 is a cell surface antigen and the first identified member of a class of pentaspan membrane proteins [41], serving as a putative stem cell marker in both hematopoietic and non-hematopoietic tissues [42]. It is involved in signaling pathways that are associated with cell proliferation, differentiation, and apoptosis. Moreover, its interaction with Wnt and Notch pathways is well established [43], while CD133 expression is also associated with reduced DNA repair capacity [44]. It is commonly used as a marker for CSCs in lung cancer and in various cancer types. Studies have shown that CD133-positive patients with colorectal cancer [45], gastric adenocarcinoma [46], and glioma [47] present significantly poorer prognoses compared to CD133-negative individuals. Similar findings have been reported for patients with NSCLC [48]. CD133 serves as a valuable target in NSCLC treatment, as therapies like TClC can inhibit CD133-positive stem-like cells, thereby reducing tumor growth, EMT, migration, invasion, and oncogenic factor secretion, ultimately disrupting tumor progression. Additionally, CD133+ cells demonstrate heightened sensitivity to combination therapies, such as Pa + 4Mu or cisplatin + 4Mu, which significantly impair their clonogenic and spheroid-forming abilities, highlighting the potential of targeting CD133 to eliminate cancer stem-like cells [49,50].

### 5.3. Ubiquitin-Specific Protease 22 (USP22)

USP22 is a member of the deubiquitinating enzyme family, which plays a pivotal role in the protein stability regulation in removing ubiquitin moieties from target proteins. In NSCLC, USP22 has attracted attention for its potential oncogenic properties. USP22 is often overexpressed in various cancers, including NSCLC, where it may contribute to tumor progression by stabilizing oncogenic proteins and promoting cell proliferation. USP22 regulates key signaling pathways associated with cancer development and progression in NSCLC, such as the Wnt/β-catenin and PI3K/Akt pathways. Elevated levels of USP22 in NSCLC have been associated with poor prognosis, as they may facilitate tumor cell survival and resistance to apoptosis [51]. Transcription factors AP2a and AP2b are important to drive the expression of the USP22 gene promoting progression of NSCLC [52]. Moreover, USP22 has been implicated in the regulation of epithelial–mesenchymal transition (EMT), a process crucial for metastasis. Given its role in tumor biology, USP22 is being explored as a potential therapeutic target, aiming to develop novel inhibitors that could enhance the effectiveness of existing treatments and improve outcomes for NSCLC patients [38].

### 5.4. CD44

CD44 is a cell surface glycoprotein involved in cell adhesion, cell migration, and interactions with the tumor microenvironment. It is detectable in NSCLC tissue by IHC, and its expression is often enriched in CSC populations. High CD44 expression constitutes a prognostic factor, and it is associated with a more aggressive disease phenotype, increased metastatic potential, and poor prognosis in NSCLC patients [53]. Given that CD44 expression is implicated in various solid tumors, including NSCLC, targeting CD44 with monoclonal antibodies like C44Mab-3 may offer a potential therapeutic approach. Further studies are necessary to evaluate its efficacy and safety profile in NSCLC while minimizing off-target toxicities [54].

### 5.5. SOX2

SOX2 is a transcription factor with a critical role in maintaining stemness and self-renewal in stem cells. It can be identified in NSCLC tissue via IHC or immunofluorescence while its overexpression in NSCLC is linked to aggressiveness of tumor, therapy resistance, and poor prognosis [55].

### 5.6. NANOG

NANOG is a transcription factor, as well. It contributes to maintaining pluripotency and self-renewal in embryonic and adult stem cells. NANOG-positive cells in NSCLC, detected by IHC, are often in conjunction with other stem cell markers. High NANOG expression is often associated with advanced disease, therapy resistance, and poor patient outcomes [56,57].

Findings from our research group showed that the A549 (NSCLC) cell line that survived three intervals of an irradiation–recovery phase demonstrated stemness potential leading to radioresistance and cell regrowth, which in clinical terms is known as relapse. Regarding this radioresistant population of lung cancer cells, an increase in protein and gene expression levels was observed in critical cancer stem cell markers such as CD44, CD133, OCT4, SOX2, and NANOG, further elucidating the detrimental role of CSCs in anticancer therapy [58].

## 6. Therapeutic Management of Non-Small Cell Lung Cancer

**Figure 2:**
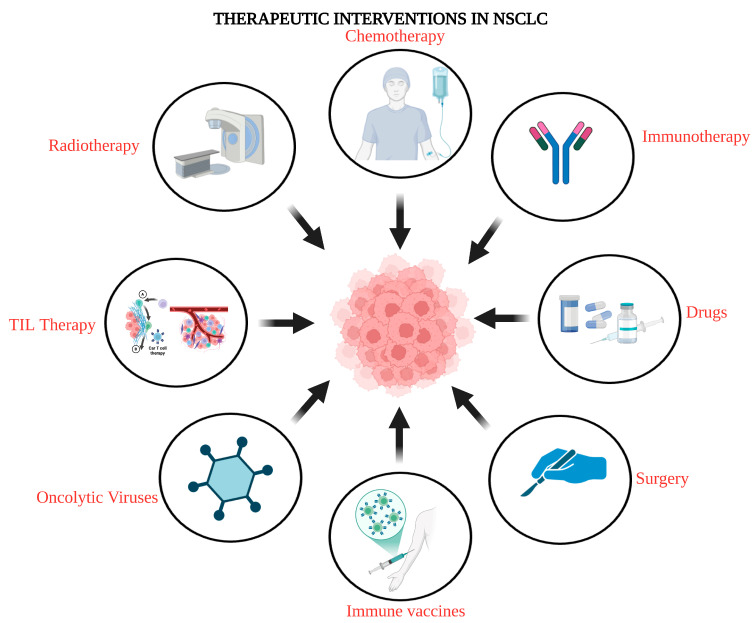
A schematic overview of innovative therapeutic interventions in NSCLC.

Non-small cell lung cancer (NSCLC) can be treated using different methods tailored to each patient’s needs and circumstances. The therapeutic plan is collaboratively developed by the patient and their cancer care team, taking into account factors such as the cancer’s stage, the patient’s overall health, and their personal preferences. A comprehensive plan outlines details about the cancer, treatment goals, available options, potential side effects, and the expected duration of therapy. Common treatment approaches include surgery, radiation therapy, and chemotherapy. The therapeutic landscape for NSCLC has undergone significant transformation in recent years, driven by new findings in the targeted therapy, and newer methods like photodynamic therapy, cryosurgery, electrocautery, and immunotherapy (Figure 2) [59].

Surgery, usually, is a primary treatment for NSCLC and different techniques depending on the extent and stage of the cancer are involved. Procedures range from wedge resection, which removes the tumor and surrounding tissue, to pneumonectomy, which removes an entire lung. Lobectomy and sleeve resection are other options that target specific lung sections or the bronchus. Post-surgical adjuvant therapy, such as chemotherapy or radiation, is often employed to eradicate any remaining cancer cells and reduce recurrence risk. Radiation therapy, another widely used treatment, employs external or internal methods to destroy cancer cells, with advanced techniques like stereotactic radiosurgery targeting tumors with precision to spare healthy tissue [60]. Depending on stage and specific characteristics of tumor, different radiation therapy schemes are used against NSCLC. For example, postoperative radiotherapy typically involved doses 40–60 Gy delivered in 25–30 fractions. In contrast, consolidation radiotherapy schemes may vary on the clinical context. Lower doses of radiation are used for palliative radiotherapy to alleviate symptoms and improve quality of patients’ life [61].

Chemotherapy and targeted therapy offer systemic treatment options for NSCLC, reaching cancer cells throughout the body. Chemotherapy involves drugs like carboplatin, cisplatin, and paclitaxel, often used alone or in combination. Innovative treatments like photodynamic therapy use light-activated drugs to destroy tumors with minimal damage to healthy tissue [62,63,64], while cryosurgery (or cryoablation) [65] and electrocautery focus on physically eliminating abnormal tissues [66], particularly in airways. Cryosurgery could improve the patients’ symptoms and quality of life after first-line chemotherapy failure [67]. Each treatment option is selected based on cancer type, location, and patient-specific factors, ensuring a tailored approach to fighting the disease.

Beyond traditional approaches, targeted therapies are the cornerstone of treatment for patients with advanced NSCLC, through drugs targeting specific genetic mutations. Drugs targeting the EGFR mutations (such as Osimertinib and Gefitinib/Erlotinib) [68], ALK inhibitor (such as Alectinib) [69], and ROS1 inhibitors (such as Crizotinib) [70] are commonly used as a first-line treatment. These therapies have demonstrated improved response rates and overall survival [71,72]. Moreover, Adagrasib and Sotorasib inhibit KRAS G12C mutations, leading to the blockage of cancer cell growth in patients with a specific genetic mutation. Drugs targeting angiogenesis, such as bevacizumab, also play a pivotal role by disrupting the tumor’s blood supply and are often used in combination with chemotherapy [73].

Another approach to treating NSCLC is vaccine therapy, which enhances anti-tumor immune responses, often in combination with immunoadjuvants [74]. For example, NEO-PV-01, a personalized vaccine using peptides from a patient’s mutated tumor DNA, demonstrated tolerable safety and induced neoantigen-specific T-cell responses in a phase I trial when combined with chemotherapy and immunotherapy [75], OSE2101, designed to target five frequently overexpressed tumor-associated antigens in NSCLC (HER-2/neu, CEA, MAGE 2, MAGE 3, and p53), showed improved overall survival in a resistant subgroup during interim analysis, with further phase III trials underway [76] and CIMAvax-EGF, a vaccine combining EGF and an immunoadjuvant, achieved a 47.6% disease control rate in a phase II trial and is now being investigated as maintenance therapy with immunotherapy in PD1-naive NSCLC patients [77]. Finally, another innovative approach, BNT116, an RNA-lipoplex vaccine encoding six TAAs, has demonstrated a tolerable safety profile in early trials and is under exploration as a combination therapy for advanced NSCLC. These developments highlight the potential of vaccines to complement existing treatments and improve outcomes for NSCLC patients [78,79].

Additionally, Oncolytic Viruses (OVs) represent an innovative approach to treating NSCLC, due to their ability to selectively lyse tumor cells. These viruses, either naturally occurring or genetically modified, also modify the tumor microenvironment to enhance anti-tumor immunity [80]. Although OVs approach, as monotherapy, has limited efficacy, combination strategies, especially with checkpoint inhibitors, hold promise. For example, Coxsackievirus A21 (Cavatak) demonstrated safety and immune activation in trials but showed modest efficacy when combined with pembrolizumab [81]. Similarly, adenovirus-based therapies, such as ADV/HSV-tk combined with radiation therapy, achieved clinical benefit in both PD-1-naive and PD-1-resistant NSCLC [82]. MEM-288, a conditionally replicative adenovirus expressing IFNβ and CD40L, showed tumor shrinkage and immune activation in early trials, with ongoing studies exploring its combination with anti-PD1 immunotherapy. These developments underscore the potential of OVs, especially in combination therapies, to improve outcomes for patients with NSCLC [83].

Tumor-infiltrating lymphocytes (TILs) and other cellular therapies represent a promising frontier in combating NSCLC. TIL therapy involves isolating, expanding, and reintroducing tumor-specific T cells into patients after lymphodepleting chemotherapy and IL2 administration to promote cell growth [84]. Despite challenges such as the need for fresh tissue and delayed treatment onset, early trials, like lifileucel (LN-145), have shown responses even in tumors resistant to immunotherapy [85]. Emerging approaches include ATL001, an autologous clonal neoantigen-reactive T cell (cNET) therapy, and gene-editing techniques like CRISPR and transcription activator-like effector nucleases (TALEN) to enhance TIL efficacy [86]. T-cell receptor (TCR) therapies, like afamitresgene autoleucel, showed encouraging responses but with manageable side effects [87]. Chimeric antigen receptor (CAR) T-cell therapy targets proteins like EGFR, though challenges like tumor antigen heterogeneity and T-cell exhaustion remain. Novel designs aim to address these limitations [88]. CAR NK cell therapies, leveraging natural killer cells, show potential for overcoming immune suppression and are being investigated in trials targeting NSCLC. These innovative therapies underscore the need for biomarker integration and advanced engineering to improve outcomes in refractory NSCLC [89].

Furthermore, the different types of immunotherapies, particularly anti-PD1/anti-PD-L1 immune checkpoint inhibitors, have revolutionized the management of NSCLC, offering new effective therapeutic options for patients with previously limited treatment options. The combination of radiotherapy and chemotherapy with immunotherapy is a well-established approach that has improved the outcome of patients with advanced NSCLC [90,91].

In addition to the aforementioned anticancer modalities, immune checkpoint inhibitors (ICIs) hold a critical role in guiding the management of advanced NSCLC, despite their relatively recent utilization. Currently, clinicians possess a variety of treatment choices, including PD-1 inhibitor monotherapy and combinations of PD-1/PD-L1 inhibitors with other traditional therapies. These combined therapies have enabled a growing number of patients to achieve durable responses.

Immune checkpoint proteins, including PD-1 and CTLA-4, have emerged as promising immunotherapy targets, significantly enhancing clinical outcomes for non-small cell lung cancer (NSCLC) patients. Currently, Pembrolizumab, an anti-PD-1 agent, is approved for use as both first-line and second-line treatment in advanced NSCLC patients whose tumors show PD-L1 expression in immunohistochemical analysis [92]. Other immunotherapy agents such as Nivolumab (anti-PD-1) and Atezolizumab (anti-PD-L1) are approved for use as second-line treatments, irrespective of PD-L1 expression [93]. Another ICI, such as Durvalumab (anti-PD-L1) is approved as a maintenance treatment for patients with inoperable stage 3 NSCLC whose disease had not progressed upon concurrent platinum-based chemoradiotherapy [94].

Recent studies have further evaluated the role of immunotherapy in previously untreated metastatic NSCLC (including both squamous and non-squamous histological types). Four major studies demonstrated an overall survival benefit upon adding PD-1 or PD-L1 inhibitors to standard chemotherapy (IMpower150 [95], IMpower130 [96], KEYNOTE-407 [97,98], KEYNOTE-189 [99]) Additionally, there are non-chemotherapy-based treatments, approaches that reduce reliance on chemotherapy and its side effects, where PD-1 or PD-L1 inhibitors are used either as monotherapy or paired with a CTLA-4 inhibitor. Findings from these studies demonstrated improved survival outcomes in biomarker-positive, NSCLC patients that have not received prior treatment (CheckMate227 [100], KEYNOTE-042 [101], KEYNOTE-024 [102]). Therefore, it is recommended that treatment-naïve metastatic NSCLC patients are likely to benefit more from first-line treatment with immunotherapy, either alone or in combination with chemotherapy. It is important to note, however, that first-line immunotherapy is not recommended for patient groups with genomic-driven lung cancers, such as those with EGFR mutations or ALK-positive NSCLC [103].

However, several clinical trials have evaluated immune checkpoint inhibitors in advanced NSCLC, including CheckMate026 trial, a phase III study comparing nivolumab to platinum-based chemotherapy in treatment-naïve advanced NSCLC patients with low PD-L1 expression levels failed to demonstrate clinical benefit [104]. Similarly, the MYSTIC trial failed to demonstrate improved progression-free survival with durvalumab as monotherapy or its combination with tremelimumab compared to platinum-doublet chemotherapy in patients with PD-L1 expression levels of less than ≥25 [105]. As a result, pembrolizumab remains the only FDA-approved single-agent immune checkpoint inhibitor (ICI) for first-line treatment in advanced NSCLC patients.

Moreover, it is important to mention that the role of PD-1/PD-L1 inhibitors in oncogene-addicted NSCLC remains uncertain, as tyrosine kinase inhibitors (TKIs) are currently the standard of care for patients with EGFR or ALK alterations. Although combining targeted therapies with PD-1/PD-L1 inhibitors has been explored, several trials were prematurely terminated due to significant toxicity reports, lack of efficacy, or other safety concerns [106,107]. As a result, immune checkpoint inhibitors (ICIs) are currently not regarded as effective for treating oncogene-addicted NSCLC. Furthermore, the high incidence of severe toxicities observed with TKIs, and immunotherapy combinations, highlights the need for a cautious and carefully monitored approach in their future development.

To maximize the benefits of immunotherapy, more effective predictive biomarkers are urgently needed. Additionally, further research is essential to understand the mechanisms underlying resistance to ICIs and to identify strategies to overcome it. In conclusion, ICIs have undeniably revolutionized the treatment of advanced NSCLC. Expanding the therapeutic benefits to a broader patient population, while addressing drug resistance and relapse factors will definitely require deeper insights into the mechanisms driving effective anti-tumor responses. The development of novel combination strategies is expected to pave the way for the next breakthroughs in cancer immunotherapy.

In the context of targeted therapies and immunotherapy, the role of personalized medicine has become increasingly attractive in the management of NSCLC. Comprehensive genomic profiling allows the identification of key mutations that could guide treatment decisions, ensuring that patients receive the most effective therapies tailored to their specific tumor characteristics [108]. Moreover, the regulation and modification of specific intracellular procedures, like autophagy [109] or ferroptosis [110], may play a crucial role to battle NSCLC. Moreover, novel therapeutic strategies, for example, gene therapy and combinations, are under investigation and seem to be promising for enhancing the overall treatment efficacy. This multidirectional approach emphasizes the importance of a comprehensive treatment strategy that involves various methods to tackle/address the complexities of NSCLC [111].

Despite the progress in therapeutic approaches, the management of NSCLC continues to be challenging, particularly in overcoming treatment resistance and identifying reliable biomarkers for predicting patient responses. Combining traditional chemotherapy with newer treatments, such as angiogenesis inhibitors and immunotherapies, is an area of active investigation [112,113]. Both inherent and acquired resistance to targeted therapies and immunotherapies remain major obstacles to achieving long-term efficacy [114]. Ongoing research is focused on the discovery of innovative biomarkers to enhance the ability to predict therapeutic outcomes, which is vital for refining treatment plans and improving survival rates [115]. Addressing these issues is essential for advancing the prognosis of NSCLC patients and maximizing the impact of available therapies.

## 7. Mutations, Tumor Microenvironment, and the Efficacy of Immunotherapy

The efficacy of immunotherapy in lung cancer is significantly affected by mutations within tumor cells, particularly those affecting tumor mutational burden (TMB) and the presence of neoantigens. Tumors with elevated TMB are more likely to generate neoantigens, which can activate T-cell responses against cancer cells. Research studies indicate that a higher number of non-synonymous mutations is linked to better clinical outcomes in patients receiving ICIs, as these mutations lead to the production of recognizable neoantigens by the immune system. For instance, Rizvi et al. has demonstrated that TMB serves as a robust indicator of response to a PD-1 blockade in NSCLC, with higher TMB associated with improved objective response rates and progression-free survival [105].

Mutation in the TP53 gene is particularly noteworthy, as it is presence in over 50% of lung cancer cases. It is observed that this mutation contributes to genetic instability, leading to an increased number of mutations and the formation of neoantigens, which enhance the immunogenicity of cancer cells and improve their response to immunotherapy [116]. Additionally, mutations in other genes, such as KRAS and STK11, have been shown to modulate immune responses and influence the sensitivity to immune checkpoint inhibitors (ICIs) [117]. Although the TP53 mutations are associated with a favorable response to ICIs, EGFR and STK11 mutations correlate with poor outcomes, underscoring the complex interplay of genetic alterations in determining treatment efficacy [118,119]. Epidermal growth factor receptor (EGFR) mutations are common in patients with advanced NSCLC and are recognized as critical oncogenic drivers of tumor growth and progression. EGFR-tyrosine kinase inhibitors (TKIs) are a class of drugs that specifically target the EGFR tyrosine kinase activity, showing significant clinical efficacy, and they are now considered as the standard of care for NSCLC patients with EGFR mutations [120,121,122,123]. However, nearly all patients acquire resistance to these EGFR-TK-inhibitors, pointing out the need for alternative therapeutic approaches [124]. Immune checkpoint inhibitors (ICIs) have gained attention as a treatment strategy in lung cancer, showing the potential for positive responses. Initial in vitro and in vivo studies showed that active EGFR oncogenic signaling induces the expression of PD-L1 and inflammatory cytokines, creating an immunosuppressive TME. They also demonstrated that anti-PD-1 treatment can reverse this effect, improving the survival of EGFR-mutant murine models [125,126]. Unfortunately, these initial preclinical positive results did not translate into clinical practice. ICIs have shown minimal efficacy in EGFR-mutant NSCLC across various trials, with low overall response rates (ORR), progression-free survival (PFS), and no significant survival benefit compared to chemotherapy [92,93,127,128]. Similar results showed clinical trials testing the combination of anti-PD-1 therapy plus chemotherapy versus chemotherapy alone. Both CheckMate 722 and KEYNOTE-789 trials showed no significant PFS improvement and no significant OS benefit from the ICI plus chemotherapy treatment [129,130].

Interestingly, adding anti-VEGF agents to the ICI-chemotherapy treatment has shown promising results for patients with EGFR-TKI-resistant NSCLC [131,132]. VEGF has been associated with reduced number of infiltrating CD8 lymphocytes either through the induction of FASL, via NFkB, or through abrupt and abnormal vascularization [133,134].

Additionally, the tumor microenvironment (TME) also plays a crucial role in the effectiveness of immunotherapy. The presence of immune suppressive cells, such as myeloid-derived suppressor cells (MDSCs), can inhibit T-cell infiltration and activity, thereby reducing the efficacy of immunotherapy [135]. In particular, the co-occurrence of mutations such as KRAS and LKB1 has been linked to a more immunosuppressive TME, leading to primary resistance to PD-1-based therapies. This suggests that not only do specific mutations influence the immunogenicity of tumors, but they also shape the TME in ways that can either promote or hinder the effectiveness of immunotherapy.

The PI3K/AKT signaling pathway holds significant importance in NSCLC as it has been implicated in tumor development, therapeutic resistance, and disease advancement. It is initiated by Receptor Tyrosine Kinases (RTKs) that respond to external signals, such as growth factors. Upon activation, RTKs stimulate the PI3K, which converts PIP2 into PIP3. PIP3 serves as a docking station on the plasma membrane, where AKT and its activator protein-kinase PDK1 bind, leading to the subsequent activation of AKT. Upon activation, AKT phosphorylates multiple substrates that play crucial roles in diverse cellular functions. This process influences cell survival by inhibiting pro-apoptotic factors such as BCL2-associated agonist of cell death protein (BAD) and caspase-9, promoting cell proliferation by facilitating cell cycle progression through the activation of mTOR and ribosomal S6 kinase (S6K), and enhancing metabolism by improving glucose metabolism and lipid synthesis [136,137].

Commonly observed mutations and changes in the components of this pathway, such as PIK3CA mutations and the inactivation of the tumor suppressor PTEN, frequently occur in NSCLC. These modifications can result in unchecked cellular growth and survival. Additionally, the pathway is linked to resistance against various cancer treatments, including chemotherapy and targeted therapies like Epidermal Growth Factor Receptor (EGFR) inhibitors. The activation of the PI3K/AKT pathway has been identified as a factor contributing to the resistance against EGFR inhibitors in NSCLC, highlighting its significance as a target for treatment approaches. Furthermore, alterations within the PI3K/AKT pathway may act as prognostic biomarkers, influencing treatment strategies and the design of clinical trials. The existence of PIK3CA mutations or the absence of PTEN expression has been associated with unfavorable outcomes in NSCLC [138,139,140].

Hyperactivation of AKT leads to an increased expression of glycolytic enzymes, such as hexokinase and PFK1, as well as the glucose transporters like GLUT1, thereby promoting anaerobic glycolysis, commonly referred to as the Warburg effect. This metabolic shift toward glycolysis results in heightened lactic acid production, which accumulates within the TME. The export of lactate and protons through transporters such as MCT1 and MCT4 contributes to the acidification of the extracellular environment, leading to acidosis in the TME. The acidic milieu adversely affects the functionality of cytotoxic T cells and natural killer (NK) cells, while simultaneously facilitating the recruitment of immunosuppressive cells, including regulatory T cells (Tregs) and myeloid-derived suppressor cells (MDSCs) [141]. Furthermore, this environment promotes tumor invasion and metastasis by aiding in the remodeling of the extracellular matrix. Consequently, the TME becomes a detrimental setting for immune cells, as the acidic conditions hinder their function by diminishing cytokine production, such as IFN-γ from T cells, impairing T-cell receptor (TCR) signaling, and inducing exhaustion or apoptosis in effector immune cells. Through these mechanisms, tumors effectively exploit the hostile TME to evade immune surveillance, often by upregulating immune checkpoint molecules like PD-L1 [142,143,144].

Targeting the AKT signaling pathway or the metabolic enzymes associated with glycolysis may help to counteract acidosis and enhance the functionality of immune cells [145]. Potential therapeutic approaches include the application of PI3K/AKT/mTOR inhibitors, such as Alpelisib and Everolimus, as well as lactate dehydrogenase inhibitors to diminish lactate production, and buffering agents like bicarbonate to mitigate the acidity of the tumor microenvironment (TME) [146,147,148].

Directly targeting AKT has also been a significant area of interest, with compounds such as Ipatasertib and Capivasertib undergoing evaluation in clinical trials. The integration of PI3K/AKT inhibitors with alternative treatment modalities, such as immunotherapy, MEK inhibitors, or chemotherapy, represents a promising strategy to improve therapeutic results in NSCLC [149,150,151,152,153]. By comprehending the relationship between AKT activation, glycolysis, and the immune response, researchers can formulate strategies to address tumor-induced immune suppression and improve the efficacy of cancer immunotherapy [146,147,148]. The PI3K/AKT signaling pathway is integral to the development and advancement of NSCLC, affecting essential processes including cell survival, proliferation, and resistance to therapy. A comprehensive understanding of this pathway provides important insights that can inform potential therapeutic approaches and contribute to the formulation of more effective treatment protocols for NSCLC. Continued research is essential to thoroughly clarify its function and enhance patient outcomes through targeted treatment options.

The TME (tumor microenvironment) is a complex network that includes fibroblasts—or cancer-associated fibroblasts (CAFs)—immune cells, endothelial cells, the extracellular matrix (ECM), and a wide range of signaling molecules that interact with cancer cells. As in most solid tumors, the TME plays a major role in NSCLC, influencing treatment effectiveness and contributing to resistance against conventional therapies, such as chemotherapy and immunotherapy. Cancer-Associated Fibroblasts (CAFs) play a crucial role, as they are integral to tumor progression, invasion, and metastasis. These cells secrete extracellular matrix (ECM) proteins and growth factors that not only facilitate tumor growth but may also contribute to resistance against therapies. CAFs are responsible for the secretion of numerous extracellular matrix components and proteases, which modify the tumor stroma in a manner that promotes tumor growth and can aid in invasion and metastasis. Additionally, they produce a variety of cytokines, chemokines, and growth factors, such as TGF-β, IL-6, and VEGF, which facilitate tumor cell proliferation, angiogenesis, and immune evasion. CAFs play a significant role in shaping the immune microenvironment by attracting immune cells and skewing them toward a protumorigenic phenotype, often resulting in immune suppression. Furthermore, they can release exosomes containing immunomodulatory molecules. Communication between CAFs and tumor cells occurs through multiple signaling pathways, frequently increasing the aggressiveness and invasiveness of the tumor. These interactions can lead to reciprocal signaling, wherein cancer cells further stimulate fibroblasts [154,155]. Furthermore, the TME is populated with a variety of immune cells, including macrophages, dendritic cells, T-cells, and myeloid-derived suppressor cells (MDSCs). The infiltration of these immune cells can have opposing effects; they may either combat tumor growth or, conversely, aid in tumor progression, contingent upon their state of activation [141,156]. Additionally, the ECM is vital, providing structural support and influencing cellular activities such as proliferation and migration. The characteristics and rigidity of the ECM can significantly impact drug delivery and therapeutic effectiveness. Various signaling molecules released by both tumor and stromal cells can alter the immune response, encourage angiogenesis, and sustain tumor growth, including pro-inflammatory cytokines such as interleukin-6 (IL-6) and tumor necrosis factor-alpha (TNF-α), as well as anti-inflammatory cytokines like interleukin-10 (IL-10) and transforming growth factor-beta (TGF-β). Additionally, various chemokines, including CCL2, CCL22, and CXCL12 (also known as SDF-1), contribute to these processes. Growth factors such as Vascular Endothelial Growth Factor (VEGF) and Fibroblast Growth Factor (FGF) are also critical. Other important components include exosomes, metabolites like Indoleamine 2,3-dioxygenase (IDO) and lactic acid, immunomodulatory molecules such as Prostaglandin E2 (PGE2) and galectins, as well as surface receptors and ligands, notably PD-L1 and CTLA-4. Moreover, abnormal blood vessel formation and function in NSCLC can restrict the delivery of oxygen and nutrients, fostering an aggressive tumor phenotype and facilitating metastatic spread [157,158,159].

The approaches for addressing the TME in NSCLC encompass the use of checkpoint inhibitors, including anti-PD-1 and anti-CTLA-4 antibodies, which can enhance the activity of T cells against tumors. Modifying the TME has the potential to increase the effectiveness of these therapeutic agents [160]. Additionally, immune cell reprogramming and the targeting of cancer-associated fibroblasts (CAFs) have emerged as promising strategies [161]. One approach to immune cell reprogramming involves converting myeloid-derived suppressor cells (MDSCs) or regulatory T cells (Tregs) into anti-tumor effector cells, achieved through ICI or cytokine therapy, such as IL-12 and GM-CSF. These cytokines facilitate the maturation of myeloid-derived suppressor cells (MDSCs) into dendritic cells or other types of antigen-presenting cells (APCs), which are proficient in activating T cell response [162,163]. Direct targeting of CAFs is another emerging area of interest. The elimination of CAFs has been shown to be promising, but there is also evidence suggesting opposing outcomes [164,165,166]. Alternatively, reprogramming CAFs has gained attention as a potential strategy. For example, in pancreatic cancer, reprogramming activated CAFs into quiescent CAFs using all-trans retinoic acid has shown promising results [167,168]. Furthermore, agents that target specific signaling pathways, such as TGF-β inhibitors, which are known to facilitate CAF activation [163], are currently under investigation [169,170,171,172].

Therapies that modify the composition and stiffness of the extracellular matrix (ECM) or inhibit particular ECM components can enhance drug delivery and lead to better therapeutic results. Enzyme therapies aimed at hyaluronan or collagen have the potential to improve anti-tumor responses [173]. Furthermore, medications that target angiogenic pathways, such as VEGF inhibitors (for instance, bevacizumab) [174,175,176], can help normalize the tumor vasculature, thereby increasing the effectiveness of concurrently administered therapies. The combination of targeted agents that address specific genetic mutations and alterations in NSCLC, along with simultaneous targeting of the TME, may yield synergistic benefits [177]. Lastly, advancements in nanotechnology facilitate the creation of drug delivery systems capable of selectively targeting TME components [178], resulting in increased drug accumulation at tumor sites and diminished systemic toxicity [170,179,180].

Numerous clinical trials are currently underway or in the planning stages, concentrating on the integration of conventional therapies—such as chemotherapy, targeted therapy, and immunotherapy—with agents specifically designed to modify the TME (KEYLYNK-006, NCT04158440) [181]. Investigating biomarkers that can predict responses to TME-targeting therapies will be crucial for tailoring treatment to individual patients. Focusing on the tumor microenvironment in NSCLC offers a comprehensive strategy aimed at improving the effectiveness of existing treatments while addressing the complexities of tumor heterogeneity and resistance. A deep understanding of the interactions within the TME will be vital for the development of novel therapeutic approaches and for enhancing patient outcomes in NSCLC. Ongoing research and clinical trials will help to elucidate the most effective methods for incorporating TME-targeting strategies into standard treatment protocols [177,182,183].

**Figure 3:**
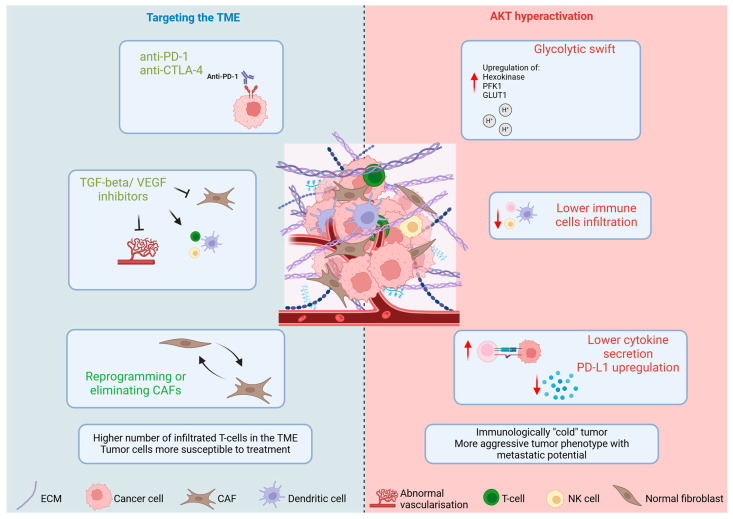
A schematic illustration of the hyperactivation axis of the AKT pathway within the framework of metabolic glycolytic shift in non-small cell lung cancer (NSCLC), and the resulting acidification of the tumor microenvironment (TME) is presented. The hyperactivation of the AKT pathway induces the Warburg effect through the upregulation of glycolytic enzymes, including hexokinase, PFK1, and GLUT1. This process leads to the secretion of protons and lactic acid by cancer cells, contributing to the acidification of the TME, which creates an inhospitable environment for the recruitment of immune cells. Additionally, the combination of diminished cytokine secretion and heightened expression of PD-L1 by cancer cells renders the tumor immunologically “cold”, characterized by an aggressive phenotype and an increased propensity for metastatic spread. The approach of targeting non-small cell lung cancer (NSCLC) through the use of monoclonal antibodies, including anti-PD-L1 and anti-CTLA4, as well as employing TGF-β/VEGF inhibitors, or by focusing on the modulation of cancer-associated fibroblasts (CAFs) with immune checkpoint inhibitors (ICIs), cytokines, or combination therapies, has the potential to modify the tumor phenotype. This strategy may result in enhanced immune cell infiltration within the tumor, decreased angiogenesis, and an increased vulnerability of tumor cells to therapeutic interventions (Image generated with BioRender.com).

In summary, mutations in lung cancer significantly affect the efficacy of immunotherapy through mechanisms involving TMB, neoantigen formation, and the modulation of the TME. The interplay of various genetic alterations, particularly those involving TP53, KRAS, and STK11, underscores the complexity of predicting immunotherapy outcomes and highlights the need for personalized treatment strategies based on individual mutational profiles (Figure 3).

## 8. Conclusions

Lung cancer remains a leading cause of cancer-related deaths, driven by complex interactions between genetic mutations, stem cell biology, and the tumor microenvironment (TME). Oncogenic mutations in genes, such as KRAS, EGFR, TP53, and STK11, play crucial roles in the transformation of normal lung stem cells into cancerous cells, leading to tumor initiation and progression. These genetic alterations disrupt normal cellular processes, enhancing proliferation, survival, and immune evasion while fostering a supportive TME. The TME, composed of immune cells, fibroblasts, and extracellular matrix components, not only sustains tumor growth but also contributes to therapy resistance, especially in non-small cell lung cancer (NSCLC). Key signaling pathways, including Wnt/β-catenin, PI3K/AKT, and Sonic Hedgehog, further augment tumor aggressiveness, underscoring the need for targeted therapeutic interventions.

Despite advancements in immunotherapy and targeted therapies, challenges such as therapy resistance and tumor heterogeneity persist, necessitating a multidimensional approach. The integration of genomic profiling with TME modulation is essential for identifying actionable biomarkers and developing personalized therapies. Moreover, the tumor microenvironment plays a crucial role in CSC regulation, supporting their survival and promoting therapy resistance. Several preclinical and clinical studies are investigating strategies to disrupt this interaction. For example, a phase I clinical trial has been initiated to evaluate the safety and preliminary efficacy of APG-2449, a novel multi-kinase inhibitor in patients with advanced solid tumors, including ALK+ NSCLC refractory to earlier-generation ALK inhibitors [184]. Additionally, innovative strategies like vaccines, combination treatments, and emerging immunotherapies hold promise for improving patient outcomes. By addressing both the genetic and microenvironmental drivers of lung cancer—particularly, the persistence of CSCs, the immunosuppressive TME, and oncogenic mutations—future research can pave the way for more effective and sustainable treatments. Strategies integrating CSC-targeted therapies, TME-modulating agents, and precision medicine approaches hold significant potential to overcome treatment resistance and improve long-term patient outcomes in NSCLC.
